# Visibility Graph Investigation of the Shallow Seismicity of Lai Chau Area (Vietnam)

**DOI:** 10.3390/e26110932

**Published:** 2024-10-31

**Authors:** Luciano Telesca, Anh Tuan Thai, Dinh Trong Cao, Thanh Hai Dang

**Affiliations:** 1 Institute of Methodologies for Environmental Analysis, National Research Council, 85050 Tito, Italy; 2 Institute of Geophysics, Vietnam Academy of Science and Technology, Hanoi 100000, Vietnam; cdtrong@igp.vast.vn (D.T.C.); dthai@igp.vast.vn (T.H.D.)

**Keywords:** reservoir-triggered seismicity, fractal, spectral, time clustering

## Abstract

In this study, the topological properties of the shallow seismicity occurring in the area around the Lai Chau hydropower plant (Vietnam) are investigated by using visibility graph (VG) analysis, a well-known method to convert time series into networks or graphs. The relationship between the seismicity and reservoir water level was analyzed using Interlayer Mutual Information (IMI) and the Frobenius norm, both applied to the corresponding VG networks. IMI was used to assess the correlation between the two variables, while the Frobenius norm was employed to estimate the time delay between them. The total seismicity, which resulted in an M≥0.8 with a *b*-value of 0.86, is characterized by a k−M slope of ≈9.1. Analyzing the variation of the seismological and topological parameters of the seismicity relative to the distance from the center of the Lai Chau reservoir revealed the following features: (1) the *b*-value fluctuates around a mean value of 1.21 at distances of up to 10–11 km, while, for distances larger than 25–30 km, it tends to the value of 0.86; (2) the maximum IMI between the monthly number of earthquakes and the monthly mean water level occurs at a distance of 9–11 km, showing a distance evolution similar to that of the *b*-value; (3) at these distances from the center of the reservoir, the time lag between the earthquake monthly counts and the monthly water level mean is 9–10 months; (4) the relationship between the *b*-value and the k−M slope suggests that the k−M slope depends on the number of earthquakes within a 22 km radius from the center of the dam. Our study’s findings offer new insights into the complex dynamics of seismicity occurring around reservoirs.

## 1. Introduction

Visibility graph (VG) analysis has become an increasingly popular statistical method for characterizing the time-dynamic properties of series across various research fields. It has been applied in economics [1,2,3,4], meteorology [5,6,7], and oceanography [8], among other fields.

By the VG method, initially introduced by Lacasa et al. [9], time series are transformed into graphs where nodes represent the values of the series, and the links or connections between nodes indicate the relationships between them. In the VG method, these connections are determined by the mutual ’visibility’ between the values of the series. The graphs obtained by the VG exhibit various parameters, with one of the most crucial being the degree, which quantifies the number of links connecting a node to others within the same graph.

In recent years, the VG method has been employed for analyzing the complexity of seismicity. Due to its ability to describe and characterize complex systems, VG analysis serves as a valuable tool for uncovering the intricate features of seismic processes. A sequence of earthquakes can be modeled as a discrete time series, where magnitudes occurring at specific times represent its values. By applying the VG to this series, the time series of magnitudes is converted into a graph.

The first analysis of the topological properties of earthquakes through the VG method was focused on the behavior of the distribution of the degree of earthquakes, whose magnitudes represented the nodes of the graph [10]. For the particular case of seismicity in Italy, it was found that this distribution was magnitude threshold independent. The relationship between the seismological and topological properties of earthquakes was firstly investigated in [11], where the *b*-value of the Gutenberg–Richter law, which approximates the frequency–magnitude distribution, was linked with the slope of the line fitting the degree versus magnitude (called the k−M slope). Successive studies performed on the real seismicity of various seismo-tectonic areas and on synthetic seismicity produced by laboratory stick–slip-type experiments reinforced such a relationship, suggesting that a certain universality might exist between the *b*-value and the k−M slope [12,13,14]. Very recently, in [15], on the basis of synthetic seismic catalogs obeying various probability distributions, it was demonstrated that the relationship between the *b*-value and the k−M slope is universal and stable, and that an estimate of the k−M slope can perform better than the commonly used *b*-value estimation methods, especially in cases with incomplete catalogs or affected by variations in the magnitude–frequency relations.

## 2. Seismo-Tectonic Settings

**Figure 1:**
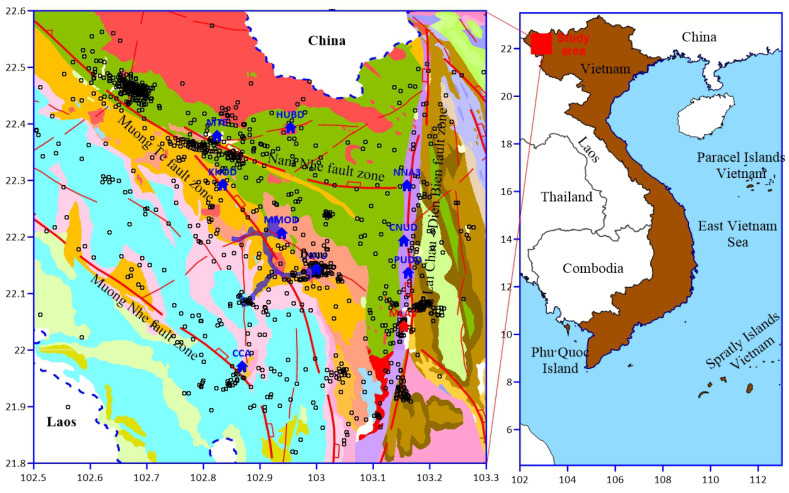
Spatial distribution of earthquake epicenters during the period of September 2014 to June 2021 (dark circles). Lai Chau reservoir is in light blue. The blue and red building symbols indicate the local seismic stations and national seismic stations, respectively. Geology and identified geologically mapped faults (red) with dip and slip direction as given by [18]. (Modified from [22]).

The collision between the Indian and Eurasian plates, which began approximately 45 million years ago, led to the formation of numerous strike–slip faults between Myanmar and the Red River fault during the early Cenozoic [16,17]. Northwestern Vietnam is a mountainous region with a complex geological structure dominated by several active faults, including the Lai Chau–Dien Bien fault zone, the Son La fault, the Ma River fault, the Da River fault, and the Red River fault. This region is the most seismically active in Vietnam, bounded by the Lai Chau–Dien Bien fault zone to the west, the Red River fault to the northeast, and the Ma River fault to the southwest. The present tectonic characteristics of these faults are determined based on surface geological investigations and studies of deep crustal structures [17,18,19,20,21]. Lai Chau Hydroelectric Reservoir is located in the northwest, with a total capacity of 1215 million $m^{3}$. Construction of the dam began on 5 November 2011, at the uppermost section of the Da River’s mainstream in Vietnam, near the border with China. The Lai Chau hydropower reservoir area has high mountainous terrain, strong cleavage, and large slopes and is strongly affected by the Muong Te fault and Dien Bien–Lai Chau fault. The geology of the area belongs to the Dien Bien Phu complex and formations such as the Da River, Nam Ma, and Nam Po (Figure 1) [18,21]. The reservoir began impounding in May 2015, and, shortly afterward, earthquakes started occurring around the dam and its vicinity. Since then, earthquakes have continued to occur, with approximately 1500 recorded to date. There are four main faults in the region under study, the Lai Chau–Dien Bien, Muong Te, Nam Nho, and Nam Nhe, with all other faults being branches of these main ones. The Lai Chau–Dien Bien fault zone is one of the most seismically active zones in Indochina, beginning in southern Yunnan Province (China) and extending over 150 km in Vietnam from the north of Chieng Chai (at the Vietnam–China border), through Lai Chau and Dien Bien Phu, to Tay Trang (near the Vietnam–Laos border). The strongest earthquakes (*M* > 5) have been recorded along this fault zone. The northern section follows a nearly north–south strike, while the southern section gradually shifts from a southward to a southwestward strike. The main fault, along with its associated subsidiary faults, is characterized by both strike–slip and oblique–slip movements, with the principal fault plane dipping westward at angles of 60–70° in the northern part and 70–80° (sometimes up to 90°) in the southern part of the fault zone [17,18,21]. During the Cenozoic, the Lai Chau–Dien Bien fault zone underwent two main phases of tectonic development, marked by dextral (right-lateral) and sinistral (left-lateral) shear activities. The shift from right-lateral to left-lateral motion occurred over 5 million years ago, during the Pliocene. The Muong Te fault zone, near to which the Lai Chau reservoir is located, is a fairly large fault running in the NW-SE direction from Chinese territory through Vietnam, with a length within Vietnamese territory of about 120 km. In the Neo-Tectonic period, the fault had two phases of deformations: the early phase of left-lateral strike–slip motion and the late phase of right-lateral strike–slip motion. Moderate earthquakes (4<M≤4.8) were observed on this fault zone. The Nam Nhe fault zone is a branch of the Muong Te fault zone that has a length < 50 km and the width of the rupture zone reaches > 1 km. Moderate earthquakes (4.1<M≤4.6) have been observed in this fault zone (Figure 1) [17,18,21]. The Muong Nhe fault zone had two phases of deformations, the same as the Muong Te fault zone.

## 3. The Frequency–Magnitude Distribution

The Gutenberg–Richter (GR) law [23] represents the relationship between the number *N* of earthquakes with magnitude $M\geq M_{th}$ and the threshold magnitude $M_{th}$. The function $log_{10}N=a-bM_{th}$ is utilized to fit the frequency–magnitude distribution of earthquakes. The parameters *a* and *b* indicate, respectively, the level of seismicity and the proportion of small events compared to large ones. The *b*-value is crucial for reliably assessing the seismic hazard of an area [24,25]. In this paper, the estimate of *b* is performed by using the maximum likelihood method (MLE) [26]:

(1) $$b=\frac{log_{10}\left(e\right)}{<M>-(M_{c}-\frac{\Delta M_{bin}}{2})}$$

where <M> is the mean magnitude of the earthquakes with magnitude $M\geq M_{c}$, $\Delta M_{bin}$ is the binning width of the catalog (generally 0.1), and $M_{c}$ is the completeness magnitude, defined as the smallest magnitude detectable by a seismic network [27].

The estimate of the *b*-value depends on the calculation of the completeness magnitude $M_{c}$. Two common methods for estimating $M_{c}$ are the goodness-of-fit (GFT) test and the maximum curvature (MAXC) method [28]. In the GFT method, $M_{c}$ is calculated by comparing the observed frequency–magnitude distribution with synthetic distributions. These synthetic distributions are generated using the values of *a* and *b* estimated from the observed data for magnitudes $M\geq M_{co}$ as a function of increasing cutoff magnitude $M_{co}$.

Let *R* represent the absolute difference in the number of earthquakes in each magnitude bin between the observed and synthetic distributions. The completeness magnitude, $M_{c}$, is determined at the first cutoff magnitude, $M_{co}$, where the observed data for $M\geq M_{co}$ can be modeled by a straight line (in a log–linear plot) for a fixed confidence level $R=R_{c}$. In the maximum curvature (MAXC) method [28], $M_{c}$ is identified as the magnitude corresponding to the largest bin in the non-cumulative frequency–magnitude distribution.

In our paper, we utilize both the MAXC and GFT methods, estimating the completeness magnitude with each. Subsequently, we determine the $M_{c}$ by selecting the maximum value between the two methods, opting for the more conservative approach.

## 4. Methods

In this study, we employed the visibility graph (VG) method, which converts a time series into a graph or network. Two arbitrary data values $\left(t_{a},y_{a}\right)$ and $\left(t_{b},y_{b}\right)$ have visibility, and, consequently, become two connected nodes of the associated graph, if any other data $\left(t_{c},y_{c}\right)$ placed between them fulfill the following [9]:

(2) $$y\left(t_{c}\right)<y\left(t_{b}\right)+\left(y\left(t_{a}\right)-y\left(t_{b}\right)\right)\frac{t_{b}-t_{c}}{t_{b}-t_{a}},$$

for the so-called Natural Visibility Graph (NVG), or

(3) $$y\left(t_{a}\right),y\left(t_{b}\right)>y\left(t_{c}\right),$$

for the so-called Horizontal Visibility Graph (HVG), where $t_{a}<t_{c}<t_{b}$.

The VG method has been applied in several research fields, like economics [1,2,3], weather forecasting [5,6], medicine [29], etc. One of the most relevant parameters of the VG method is the degree that is the number of links departing from a node; generally, the higher values of the series behave as hubs of the graph, since they “attract” more links than those converging to the lower ones.

After constructing the network associated with the time series using the visibility graph, the adjacency matrix is an N×N matrix, where *N* is the length of the time series. The element $a_{ij}$ of this matrix is equal to 1 if the nodes *i* and *j* are linked; otherwise, it is 0.

**Figure 2:**
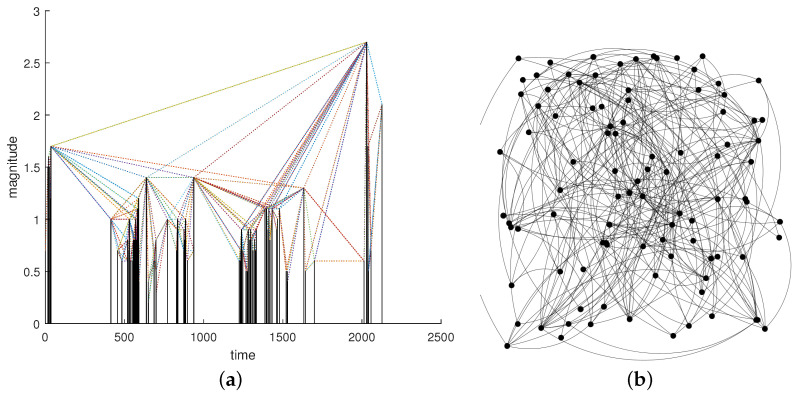
(**a**) Earthquake sequence and the links among the magnitudes defined by the VG. (**b**) Graph of the links among the nodes defined by the VG.

An earthquake sequence can be analyzed using the VG method by considering the magnitudes as the nodes of the graph and “drawing” links between two magnitudes fulfilling the “visibility” rule (Equation (2)). Thus, a seismic sequence can also be converted into a graph using the VG method. Figure 2a shows an example of an earthquake sequence with the links drawn by applying Equation (2), while Figure 2b shows the resulting graph.

The VG can also be utilized to explore the relationship between two time series by employing the *Interlayer Mutual Information* (IMI), $I_{\alpha \beta }$. Consider two time series $x_{\alpha }\left(t\right)$ and $x_{\beta }\left(t\right)$ and their VG (${\mathrm{VG}}_{\alpha }$ and ${\mathrm{VG}}_{\beta }$). Let $k_{\alpha }$ and $k_{\beta }$ denote their respective degree sequences.

The IMI is defined as follows [30]:

(4) $$I_{\alpha \beta }=\sum_{k_{\alpha }}\sum_{k_{\beta }}P\left(k_{\alpha },k_{\beta }\right)\log\left(\frac{P(k_{\alpha },k_{\beta })}{P\left(k_{\alpha }\right)P\left(k_{\beta }\right)}\right),$$

where $P(k_{\alpha },k_{\beta })$ is the joint probability of finding a node with a degree of $k_{\alpha }$ in ${\mathrm{VG}}_{\alpha }$ and $k_{\beta }$ in ${\mathrm{VG}}_{\beta }$. This joint probability is computed as follows:

(5) $$P\left(k_{\alpha },k_{\beta }\right)=\frac{N_{k_{\alpha },k_{\beta }}}{N},$$

with $N_{k_{\alpha },k_{\beta }}$ being the number of nodes that have the corresponding degree of $k_{\alpha }$ and $k_{\beta }$ in ${\mathrm{VG}}_{\alpha }$ and ${\mathrm{VG}}_{\beta }$, respectively. Since *N* is the total number of nodes, it must hold that it is equal to the sum over all possible $N_{k_{\alpha },k_{\beta }}$ values.

By definition, $I_{\alpha \beta }$ describes the the average statistical dependency between the degree sequences, which can be used to characterize the nature of the time series [9,31]. From this, it can be inferred that the higher this parameter is, the more similarly the signals underlying the VGs are expected to behave.

Recently, ref. [32] proposed a method to compare two series and identify the time lag between them using the visibility graph algorithm, which has been shown to be more effective than cross-correlogram techniques. If $A_{\alpha }$ and $A_{\beta }$ are the adjacency matrices of the VGs of time series $x_{\alpha }\left(t\right)$ and $x_{\beta }\left(t\right)$, respectively, we can define their “distance”, the quantity $∥A_{\alpha }-A_{\beta }{∥}_{2}$, where ${∥\cdot ∥}_{2}$, called the Frobenius norm of a matrix, is the square root of the sum of the squares of the elements of the matrix, that is, the square root of the trace of the product of the matrix with itself. In mathematical notation, if *D* denotes the matrix $A_{\alpha }-A_{\beta }$ and $D_{ij}$ the (i,j)th element of *D* (with i,j=1,…,T), then

(6) $$∥A_{\alpha }-A_{\beta }{∥}_{2}={∥D∥}_{2}=\sqrt{\mathrm{Trace}(DD^{T})}=\sqrt{\sum_{i=1}^{T}\sum_{j=1}^{T}D_{ij}^{2}}.$$

To calculate the time lag between $x_{\alpha }\left(t\right)$ and $x_{\beta }\left(t\right)$, convert $x_{\beta }$(t) into its corresponding adjacency matrix, $A_{\beta }$. Consider time-shifted copies of the $x_{\alpha }\left(t\right)$, ($x_{\alpha ,\tau_{1}}\left(t\right),\ldots ,x_{\alpha ,\tau_{k}}\left(t\right)$, each shifted in time by a lag from the set $\left\{\tau_{1},\ldots ,\tau_{k}\right\}$. Convert these time-shifted copies of $x_{\alpha }$ into their visibility graphs and obtain the corresponding adjacency matrices $A_{\alpha ,\tau_{1}},\ldots ,A_{\alpha ,\tau_{\kappa }}$. We determine the copy $A_{\alpha ,\tau_{s}}$ for which the Frobenius norm $∥A_{\beta }-A_{\alpha ,\tau_{s}}{∥}_{2}$ is minimized. The time lag between the two original series is then taken as $\tau_{s}$.

**Figure 3:**
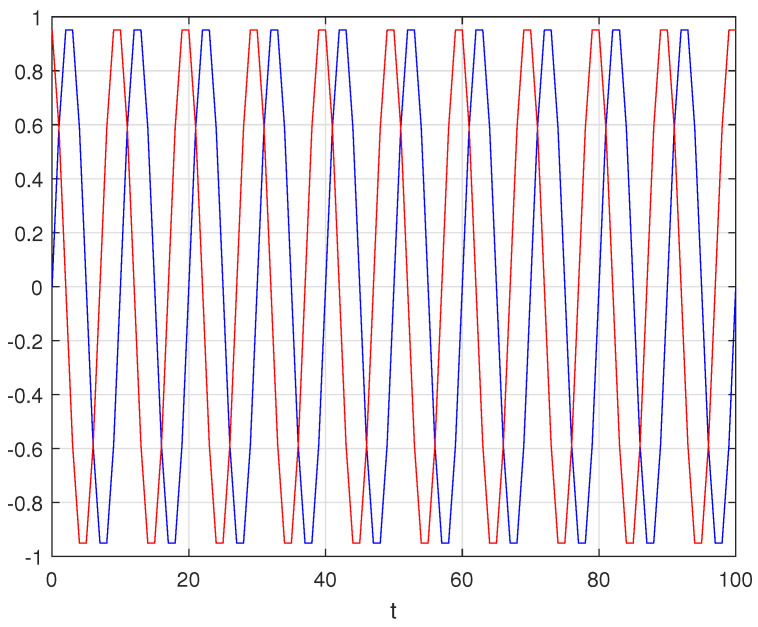
Comparison between two sinusoids, $y\left(t\right)=\sin\left(\frac{2\pi t}{10}\right)$ (blue) and $y_{d}\left(t\right)=\sin\left(\frac{2\pi (t-3)}{10}\right)$ (red), both with period T=10 and the second one delayed by d=3.

**Figure 4:**
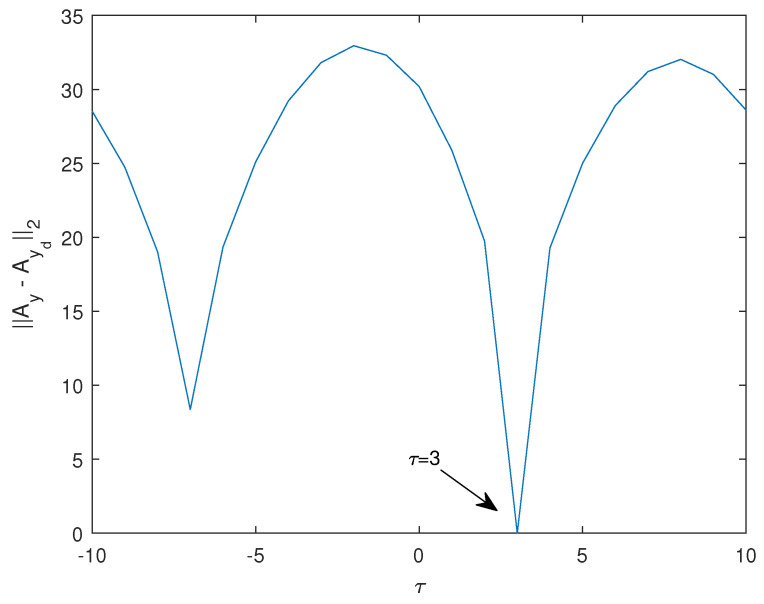
Froebenius norm $∥A_{y}-A_{y_{d}}{∥}_{2}$ between the two sinusoids plotted in Figure 3.

As an example, consider two sinusoids, $y\left(t\right)=\sin\left(\frac{2\pi t}{10}\right)$ and $y_{d}\left(t\right)=\sin\left(\frac{2\pi (t-3)}{10}\right)$. Both have a period of T=10, while the second one is delayed by d=3 (Figure 3). Plotting $∥A_{y}-A_{y_{d}}{∥}_{2}$ between the two sinusoids, the minimum is at τ=3, which is exactly the delay of the second sinusoid with respect to the first one (Figure 4).

## 5. Results

### 5.1. Analysis of the Whole Seismic Dataset

**Figure 5:**
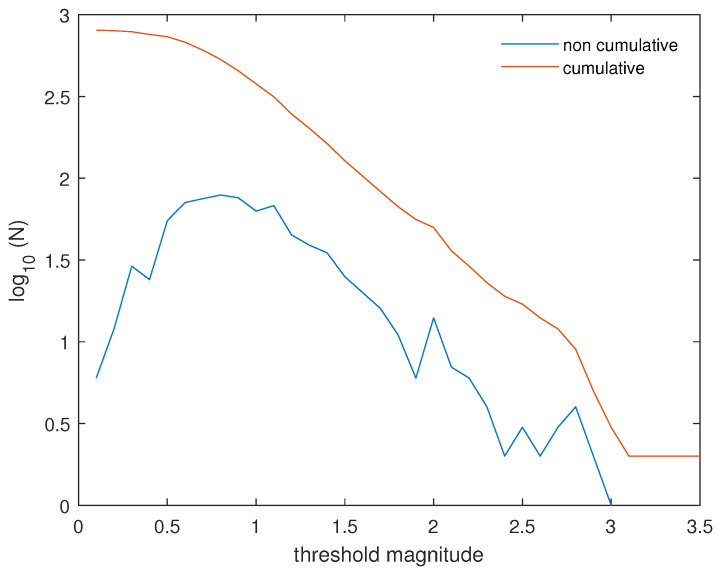
Frequency–magnitude distribution of earthquakes occurring at depths of up to 10 km and within a 40 km radius from the center of the dam.

In our study, we analyzed the shallow (depth ≤ 10 km) seismicity that occurred around the Lai Chau reservoir, up to 40 km from the center of the dam, from 2015 to 2021, and its relationship with the water level. The seismic catalog contains 805 events. Figure 5 shows the cumulative and non-cumulative frequency–magnitude distribution of the earthquakes occurring at depths of up to 10 km and within a 40 km radius from the center of the dam. Applying the MAXC and GFT methods with a confidence level of 90%, we obtained $M_{c}$ values of 0.8 and 0.7, respectively. Therefore, we selected 0.8 as the completeness magnitude for the seismic dataset. With this value of $M_{c}$, the complete seismic catalog contains 533 events. Applying Equation (1), the value of *b* is 0.86 ± 0.03.

**Figure 6:**
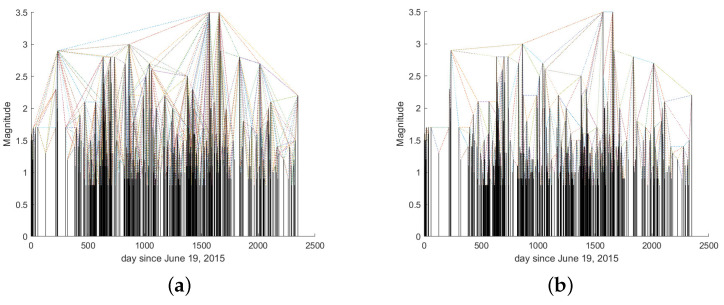
Sequence of earthquakes occurring within 40 km of the center of Lai Chau reservoir. The links among the magnitudes are defined by the NVG (**a**) and HVG (**b**).

**Figure 7:**
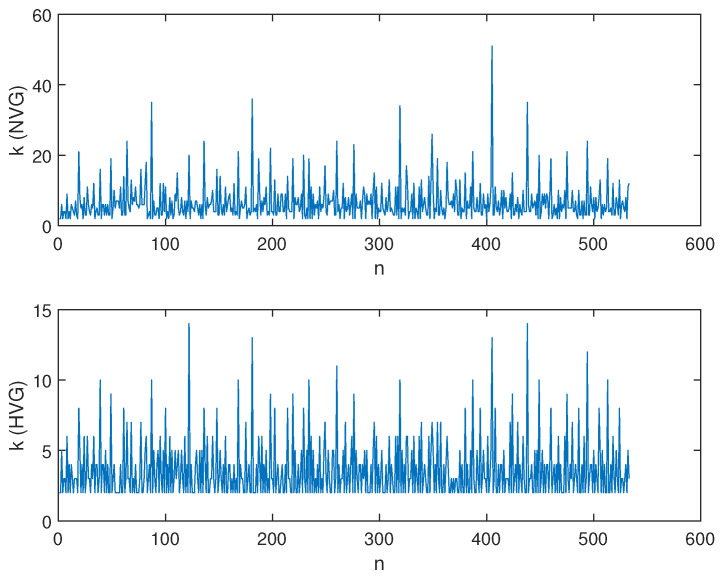
Sequences of degree *k* for the NVG and HVG applied to the seismic dataset shown in Figure 6.

We applied the VG method to this seismic dataset. Figure 6 shows the NVG and HVG applied to the seismic dataset. At each magnitude, *M* is associated with a degree *k* that represents the number of links of that magnitude (Figure 7).

In a study by Telesca et al. [11], an interesting relationship was found between the degree *k*(NVG) of a seismic sequence and magnitude. The slope of the linear fit of such a relationship is called the k−M slope. Several studies of observational [13], experimental [33], and physics-based synthetic [14] seismic catalogs have shown a positive correlation between the k−M slope and the *b*-value. Recently, ref. [15] demonstrated that the relationship between the k−M slope and *b* is universal and stable, and that their ratio depends on the size of the catalog.

**Figure 8:**
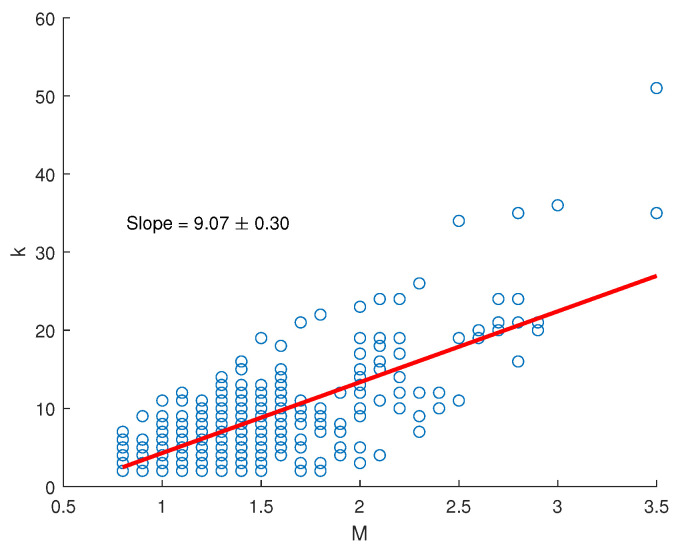
*k-M* relationship between the degree and the magnitude for the whole seismic dataset. The slope of the regression line is 9.07.

**Figure 9:**
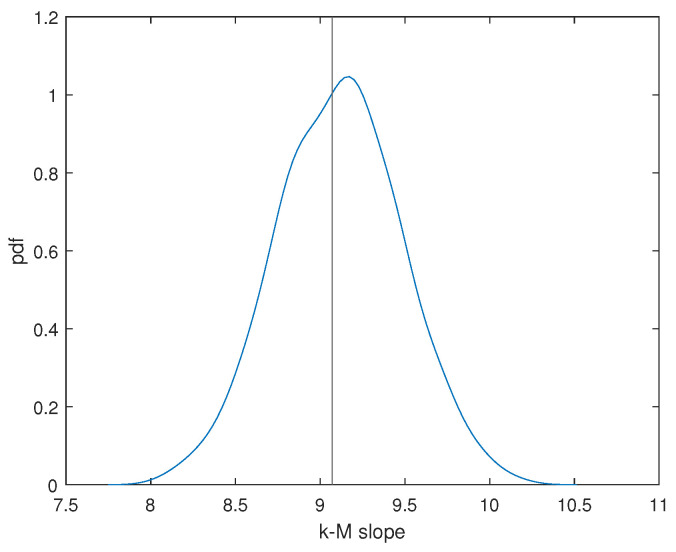
Distribution of the *k-M* slope for the randomized earthquake sequences. The vertical line indicates the *k-M* slope of the original sequence.

The relationship between the *k*(NVG) and *M* of our seismic dataset is illustrated in Figure 8, along with the fitting regression line, whose slope is the *k*-*M* slope. The k−M slope is positive (9.07±0.30), and this indicates that the degree and the magnitude are positively correlated; thus, the larger the magnitude, the higher the degree. In fact, the more intense events behave as “hubs” of the seismic sequence, attracting more connections than the smaller events and being made visible by more earthquakes than the smaller ones. We generated 1000 randomize earthquake sequences by shuffling the magnitudes while preserving the original times of occurrence. For each random sequence, we calculated the *k-M* slope. Figure 9 illustrates the distribution of the *k-M* slopes from the randomized sequences, alongside the *k-M* slope derived from the original seismic series. It is evident that the randomization does not significantly alter the *k-M* slope of the original sequence. This is due to the dominant role of the largest events, which continue to act as ’hubs’ of the sequence even after shuffling the magnitudes, thereby driving the structure of the seismic network. This suggests that the *k-M* relationship is primarily influenced by the presence of these large events.

**Figure 10:**
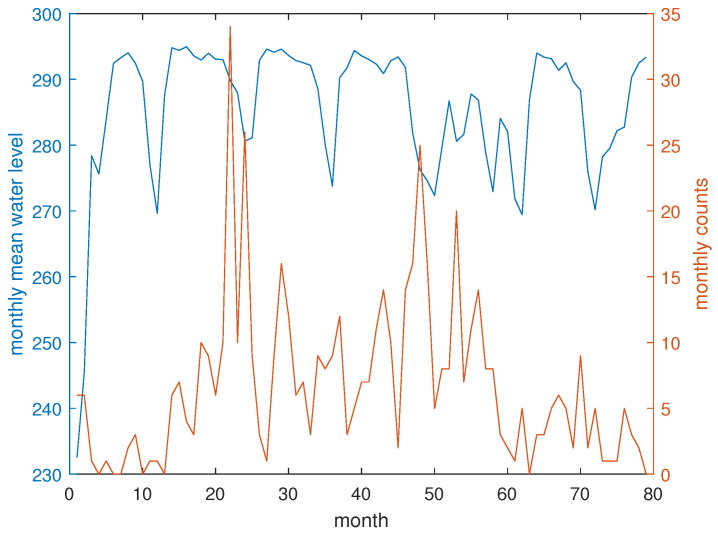
Monthly earthquake counts (red) and mean water level (blue) during the investigation period.

Figure 10 illustrates the monthly variations in the number of earthquakes (with $M\geq M_{c}$) alongside the mean water level of the Lai Chau reservoir. Applying Equation (4), the IMI calculated by using the NVG is ∼1.07 and that calculated by using the HVG is ∼0.41.

### 5.2. Distance-Dependent Analysis of Seismicity

To investigate the changes in the seismological and topological properties of the seismicity relative to the distance from the center of the dam, we analyzed seismic events occurring within a range of 6 to 40 km from the center.

**Figure 11:**
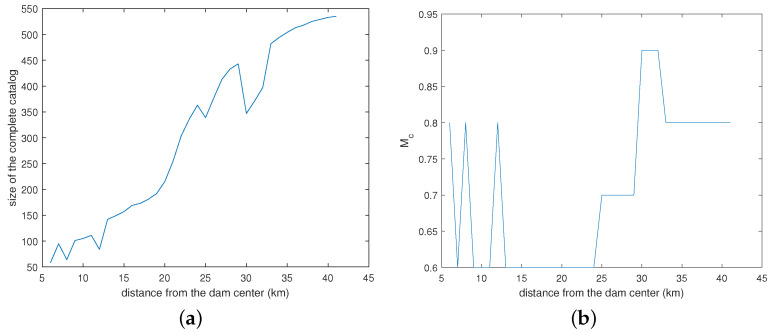
Number of earthquakes in the complete seismic catalog (**a**,**b**) showing completeness magnitude versus distance from the center of the reservoir.

Figure 11 shows the variation with the distance from the center of the completeness magnitude (Figure 11b) and the number of earthquakes with magnitude greater than or equal to $M_{c}$ (Figure 11a).

**Figure 12:**
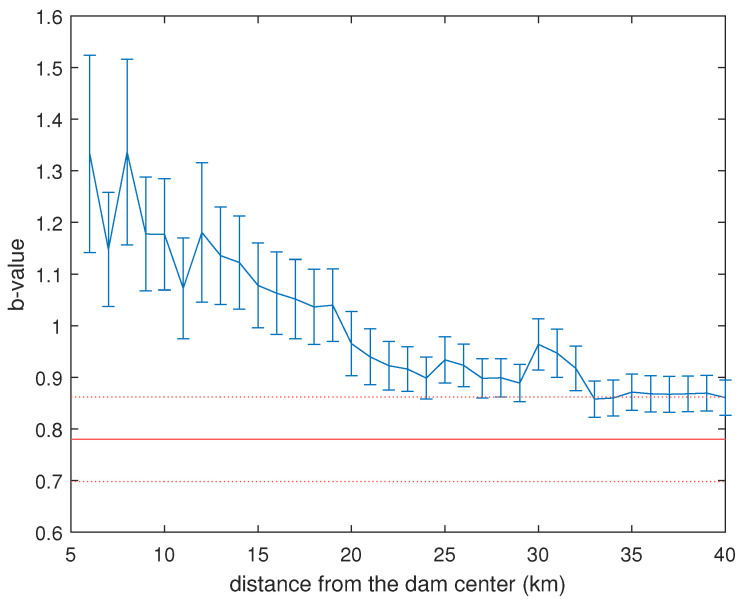
Variation of the *b*-value with the distance from the center of the dam (blue). The red horizontal line represents the *b*-value calculated for the tectonic seismicity observed prior to the reservoir impoundment, corresponding to a completeness magnitude of 0.7, as indicated in [34]. The error on *b* is indicated by the vertical bars, while the red dotted horizontal lines delimit the error band on *b* calculated in [34].

Figure 12 illustrates the variation of the *b*-value with distance from the dam center. The red horizontal line represents the *b*-value calculated for the tectonic seismicity observed prior to the reservoir impoundment, corresponding to the completeness magnitude $M_{c}=0.7$, as indicated by Lizurek et al. [34]. Three distinct regimes can be observed in the variation of the *b*-value with distance from the center of the dam:

1. Up to approximately 10–11 km, the *b*-value ranges around a mean value of ∼1.21.
2. Between approximately 10–11 km and 24–25 km from the center of the dam, the *b*-value decreases with the distance from the center of the dam.
3. For distances greater than approximately 25–30 km from the dam center, the calculated *b*-value closely matches the value reported by Lizurek et al. [34].

Since Lizurek et al. [34] calculated the *b*-value for the background seismicity that occurred before the impoundment of the reservoir, the events occurring more than ∼24–25 km from the dam are likely tectonic in nature. In the first regime (up to approximately 10–11 km), the *b*-value is significantly larger than that of the regional seismicity, which could provide additional evidence that the earthquakes occurring near the dam are likely reservoir triggered [35]. In the regime of intermediate distances from the dam center, the decrease in the *b*-value could suggest a mixture of tectonic and reservoir-triggered earthquakes, with seismicity tending to be dominated by tectonic earthquakes as the distance from the dam increases.

**Figure 13:**
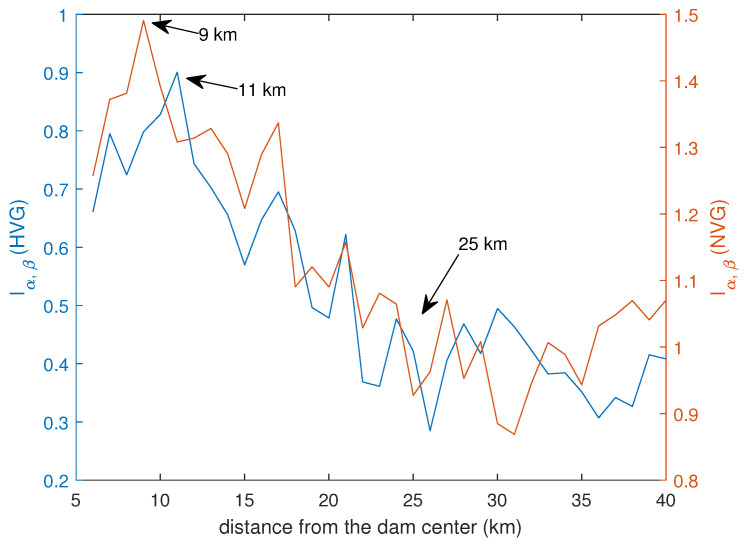
IMI between monthly number of earthquakes and monthly mean water level calculated by NVG (red) and HVG (blue).

We calculated the IMI between the monthly number of earthquakes and the monthly mean water level (Figure 13). The variation of the IMI with the distance from the center of the dam is quite similar to that of the *b*-value; for small distances, it increases, reaching a maximum at 9–11 km, then it decreases up to approximately 25 km. For larger distances, it fluctuates around mean values of approximately 0.99 (NVG) and 0.39 (HVG).

The similarity in the distance-related variation of both the *b*-value and IMI is very striking; three distance regimes are identified in the *b*-value variation, which closely match those identified in the IMI variation. The distance of 9–11 km, at which the IMI reaches its maximum, could be considered the furthest distance from the dam where the water level fluctuations impact on seismicity.

**Figure 14:**
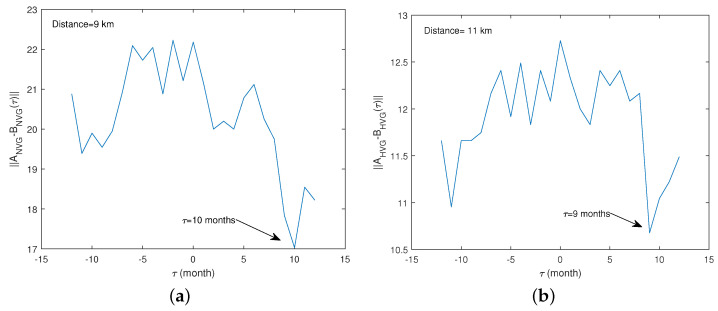
Variation with the time lag τ of the Frobenius norm of the difference between the adjacency matrix of the monthly number of earthquakes and that of the τ-shifted monthly mean of water calculated by using the NVG (**a**) and the HVG (**b**).

Considering the distance from the center of the dam corresponding to the peak of IMI, we computed the time lag between the monthly number of earthquakes and the monthly mean water level shifted by lag τ ranging from −12 to 12 months (Figure 14). In both cases, the time lag between the two variables is 9–10 months, showing that the occurrence of earthquakes follows water level fluctuations with a delay of 9–10 months.

**Figure 15:**
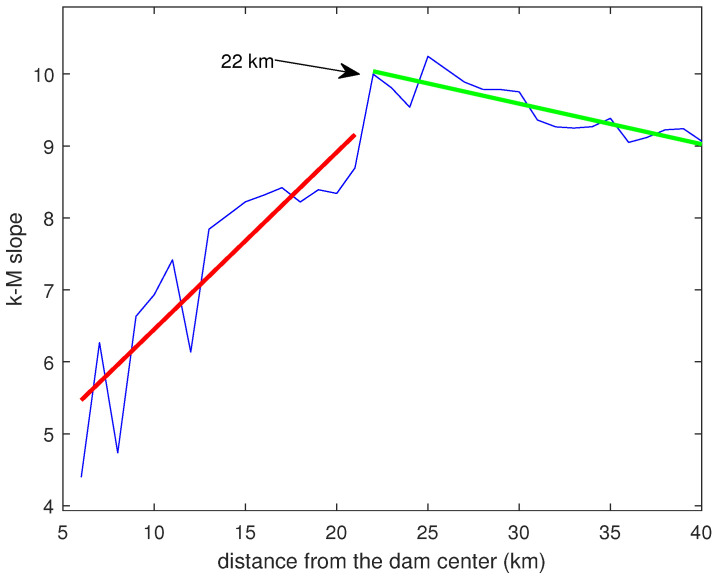
Variation of the k−M slope with the distance from the center of the dam.

**Figure 16:**
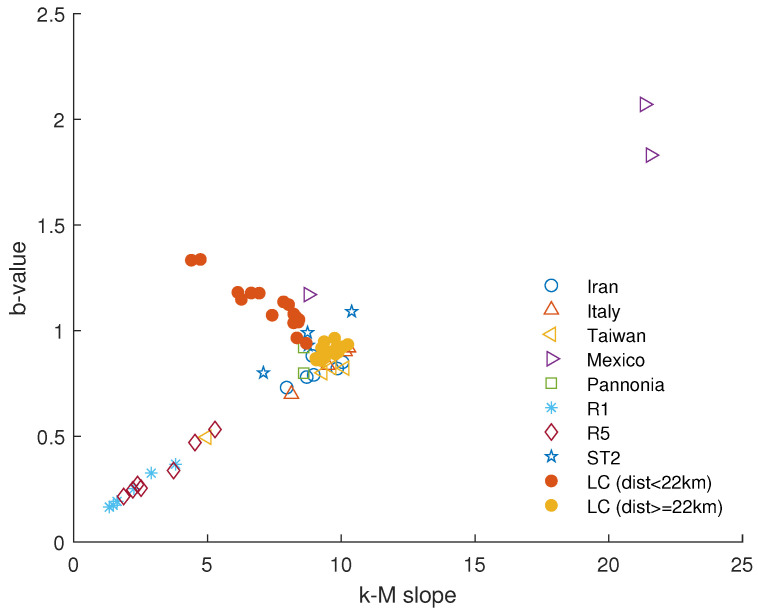
Relationship between the *k*-*M* slope and the *b*-value for the Lai Chau dataset (for distances from the dam center less than, larger than, or equal to 22 km) compared with that of the seismic datasets analyzed in previous studies (Iran [12], Italy and Taiwan [33], Mexico [11], Pannonia [38], R1 and R2 [39], and ST2 [37]).

**Figure 17:**
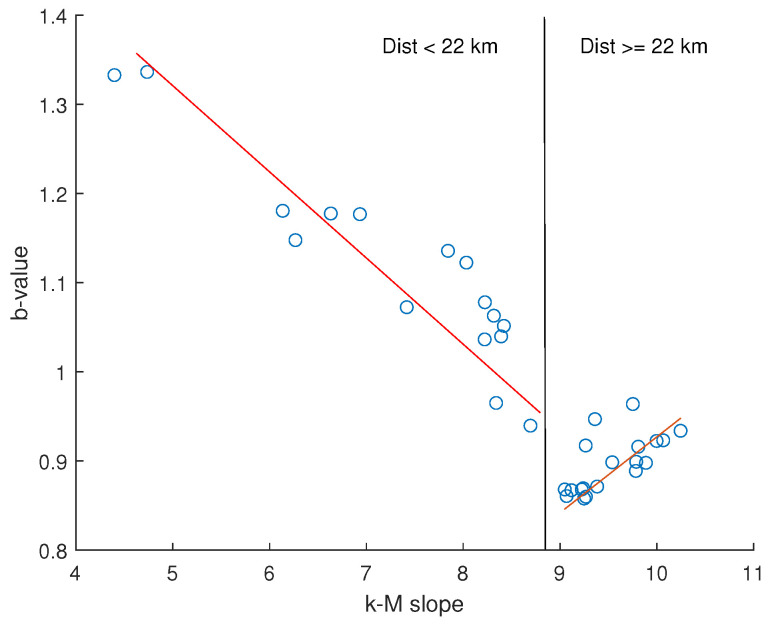
Relationship between the k−M slope and the *b*-value for the seismic datasets extracted at distances from the center varying from 6 to 40 km. The vertical black line separates the values relative to distances less than, greater than, or equal to 22 km from the center of the dam.

**Figure 18:**
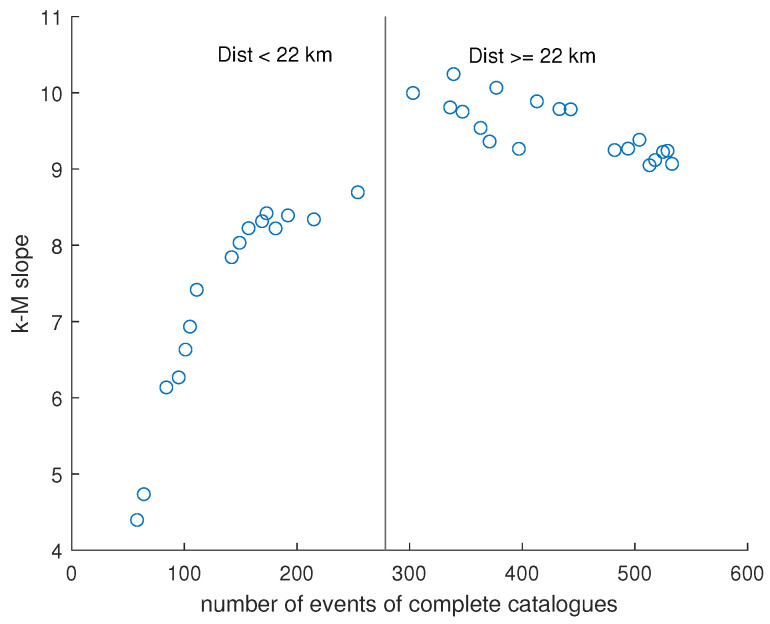
Relationship between the k−M slope and the number of events for the seismic datasets extracted at distances from the center varying from 6 to 40 km. The vertical black line separates the values relative to distances less than, greater than, or equal to 22 km from the center of the dam.

Figure 15 shows the variation of the k−M slope with the distance from the dam center. This topological parameter exhibits an increasing trend followed by an approximately constant or weakly decreasing one. In order to more precisely identify the breakpoint between these two trends, we applied the segmented regression method [36]. The found breakpoint is at 22 km. Figure 16 shows the relationship between the *b*-value and the k−M slope for the Lai Chau seismic sequence compared with that of seismic datasets analyzed in previous studies [11,12,37]. For distances from the center of the dam greater than or equal to 22 km, the Lai Chau seismic dataset aligns with the general relationship found for the other seismic datasets, while, for smaller distances, the seismic sequences in the Lai Chau area deviate from this general relationship. Focusing only on the Lai Chau dataset, the relationship between the *k*-*M* slope and the *b*-value (Figure 17) is characterized by two distinct behaviors. For distances from the dam center less than 22 km, the relationship is negative, while for distances greater than or equal to 22 km, the relationship is positive. To explain the negative relationship for distances less than 22 km, we analyzed the *k*-*M* slope in relation to the number of events for each distance. We found that, up to about 22 km from the center of the dam, the *k*-*M* slope increases with the number of events, whereas, for distances greater than 22 km, the *k*-*M* slope appears to be independent of the number of events (Figure 18).

Recently, Li et al. [15] found that the ratio between the k−M slope and the *b*-value can be influenced by the catalog size and, for Poissonian catalogs, derived the following relationship between this ratio and the number *N* of earthquakes:

(7) $$\frac{k-M\;\mathrm{slope}}{b}=-15.15{\left(\log_{10}N\right)}^{-2.14}+11.85.$$

**Figure 19:**
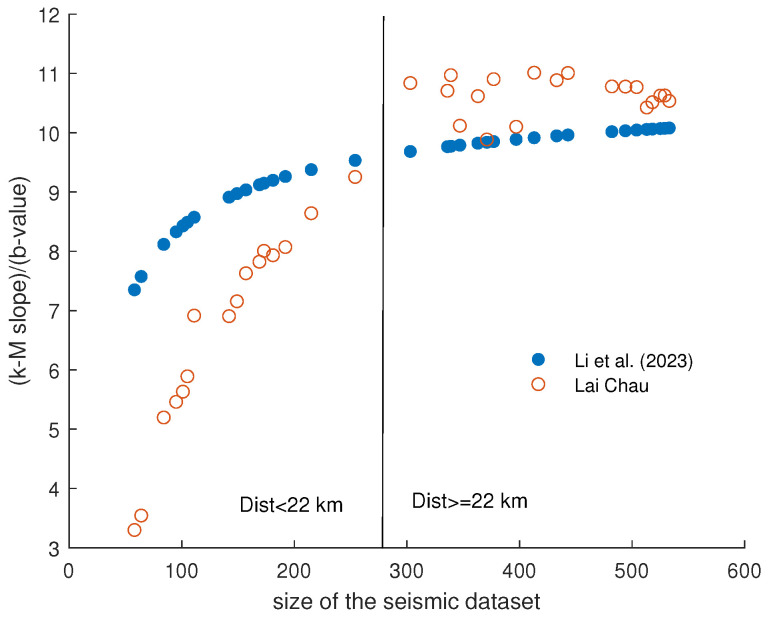
Variation of the ratio between k−M slope and the *b*-value with catalog size for Lai Chau dataset (red circles) compared with the relationship proposed in [15] (blue filled circles).

We calculated the ratio between the k−M slope and the *b*-value for the Lai Chau data and related it to the number of earthquakes, changing the distance from the dam center. The results are shown in Figure 19 and compared with Equation (7). Although the two curves do not perfectly overlap (Equation (7) is derived from Poissonian catalogs), their trends are almost identical. There is a clear increase in the ratio with the size of the catalog, which tends to stabilize as the catalog size grows. For the Lai Chau dataset, the saturation of the ratio starts for distances from the dam center larger than 22 km.

## 6. Conclusions

In this study, we analyzed the time dynamics of the shallow seismicity occurring in the vicinity of the Lai Chau reservoir (Vietnam), focusing on its seismological and topological properties, along with their mutual relationship. We employed the VG method to convert the investigated series into networks and analyzed a variety of network quantities (degree, k−M slope, IMI, and Frobenius norm) related to the earthquake series and also to the reservoir water level. The total seismicity, whose completeness magnitude is 0.8 and *b*-value is 0.86, is characterized by a k−M slope of ≈9.1. The positive value of the k−M slope indicates a positive correlation between degree and magnitude, which means that the higher the magnitude, the larger the degree. Thus, the larger events behave as “hubs” of the VG seismic network, being made visible by more earthquakes than the smaller ones. The *b*-value shows a non-trivial pattern as the distance from the dam’s center increases. For distances up to 10–11 km, it ranges around a mean value of 1.21, while, for distances larger than 25–30 km, it aligns with the background value found by [34]; for intermediate distances, the *b*-value decreases with the distance from the dam’s center. The IMI between the monthly number of earthquakes and the monthly mean water level is at its maximum at a distance of 9–11 km, and, at these distances, the time lag between the two variables is 9–10 months. The relationship between the *b*-value and the k−M slope seems to suggest that the k−M slope depends on the number of earthquakes within a 22 km radius from the center of the dam.

## Data Availability

IS EPOS (2018), Episode: LAI CHAU, https://episodesplatform.eu/#episode:LAI_CHAU, (accessed on 2 April 2024) https://doi.org/10.25171/InstGeoph_PAS_ISEPOS-2018-012, (accessed on 2 April 2024).
